# Evaluation of Polyphenolic Profile and Nutritional Value of Non-Traditional Fruit Species in the Czech Republic — A Comparative Study

**DOI:** 10.3390/molecules17088968

**Published:** 2012-07-27

**Authors:** Tunde Jurikova, Jiri Sochor, Otakar Rop, Jiří Mlček, Štefan Balla, Ladislav Szekeres, Rastislav Žitný, Ondrej Zitka, Vojtech Adam, Rene Kizek

**Affiliations:** 1 Department of Natural and Informatics Sciences, Faculty of Central European Studies, Constantine the Philosopher University in Nitra, Drazovska 4, SK-949 74 Nitra, Slovakia; Email: tjurikova@ukf.sk (T.J.); lszekeres@ukf.sk (L.S.); rzitny@ukf.sk (R.Ž.); 2 Karel Englis College, Sujanovo nam. 356/1, CZ-602 00, Brno, Czech Republic; Email: sochor.jirik@seznam.cz (J.S.); ondrej.zitka@mendelu.cz (O.Z.); vojtech.adam@mendelu.cz (V.A.); kizek@sci.muni.cz (R.K.); 3 Department of Chemistry and Biochemistry, Mendel University in Brno, Zemedelska 1, CZ-613 00 Brno, Czech Republic; 4 Central European Institute of Technology, Brno University of Technology, Technicka, 3058/10, CZ-616 00 Brno, Czech Republic; 5 Department of Food Technology and Microbiology, Faculty of Technology, Tomas Bata University in Zlin, Namesti T. G. Masaryka 275, CZ-762 72 Zlin, Czech Republic; Email: rop@ft.utb.cz (O.R.); mlcek@ft.utb.cz (J.M.)

**Keywords:** non-traditional fruit species, dry matter, organic acids, ascorbic acid, minerals, polyphenolic compounds

## Abstract

Dry matter, organic acids, ascorbic acid, minerals (nitrogen, phosphorus, potassium, calcium, magnesium, sodium) and polyphenolic profile of a number of non-traditional fruit species and their genotypes, namely blue honeysuckle (*Lonicera* spp.), Saskatoon berry (*Amelanchier alnifolia*), black mulberry (*Morus nigra*), Tomentosa cherry (*Prunus tomentosa* Thunb.) and jostaberry (*Ribes nigrum* x *Grossularia uva-crispa*) were investigated. The results showed that *Lonicera *genotypes displayed high levels of ascorbic acid and they were rich in minerals, with the cultivar ‘Amfora’ achieving the leading position in nitrogen, phosphorus and potassium content among all lesser known fruit species. *Amelanchier* cultivars represented a valuable source of ascorbic acid and calcium, ‘Tišňovský’ and ‘Smoky’ together with *Morus nigra* ‘Jugoslavska’ accumulated the highest level of examined polyphenolic compounds. Regular consumption of studied less common fruit species can bring health benefits so they can represent a high potential value for fruit growers and in addition they can be utilised as functional foods.

## 1. Introduction

Berries contain powerful antioxidants and a proper balance of bioactive compounds. They are considered to be a good source of phenolic compounds, especially flavonoids and phenolic acids, which mostly contribute to their high antioxidant activity. Berries have recently received much attention for their health benefits, including antimutagenesis and anticarcinogenic activity for the prevention of various cancers and age-related diseases [1,2,3,4]. Nowadays, increasing attention is being paid by consumers to the lesser known fruits such as Cornelian cherry, honeyberry, lingonberry, elderberry, sea buckthorn, medlar, bilberry and Saskatoon berries which have unusual flavours and qualities, and many of them are rich in antioxidants and anthocyanins [5,6]. Generally, non-traditional fruit species belong to the following groups: older fruit species like black mulberry (*Morus nigra*), well-known wild fruit species of interest for further breeding processes or are originating from breeding process like Cornelian cherry (*Cornus mas*)*, *jostaberry (*Ribes nigrum *× *Grossularia uva*-*crispa*) and finally the fourth group is represented by foreign lesser known fruit species promising because of the high nutritional value of the fruit, especially a high level of ascorbic acid and polyphenols [7]. The edible honeysuckle holds a significant position among the fourth group (*Lonicera* spp.)-species, with unique biological and chemical properties [8]. Edible honeysuckles originated in Russia and they are especially rich in ascorbic acid and polyphenolic compounds [9,10] and they display a high antioxidant activity thanks to the prevailing content of neuroprotective plant phenols (rutin, quercitrin, gallic acid and 4-aminobenzoic acid) [11,12,13] depending on the stage of ripeness [14]. The freeze-dried fruits of *Lonicera caerulea* and its phenolic fraction reduced the biofilm formation and adhesion to the artificial surface of *Candida parapsilosis*, *Staphylococcus epidermidis*, *Escherichia coli*, *Enterococcus faecalis*, and *Streptococcus mutans* [15], and exhibit antifungal [16] and anticancer properties [17].

The Saskatoon berry is a less known cultivated pomaceous fruit. The Saskatoon berry is distributed in the southern Yukon and Northwest Territories, the Canadian prairie province and northern prairies of the United States [18]. It was traditionally used as a food source by the native people and early settlers in these regions and has been cultivated and grown in orchards similar to blueberries. In Europe it is rarely grown, in spite of this, it shows many outstanding technological and nutritional parameters, especially high levels of phenolic acids and flavonoids [19,20].

Over the last two decades new cultivars were bred in the Czech Republic [21]. The current studies have showed that two cultivars of Saskatoon berries, ‘Thiessen’ and ‘Smoky’, possess a high radical scavenging activity attributed to the presence of anthocyanin in the phenolic fractions and their extracts suppressed peroxy-radical intracellular oxidation [22].

Black mulberry (*Morus nigra*) seems to be significantly effective in the reduction of blood glucose that can be supplemented in diabetes [23]. The flavonoid extract from the seeds of Tomentosa cherry (*Prunus tomentosa* Thunb.) displayed antioxidative [24] and inhibitory activities on nitric oxide (NO) and prostaglandin E-2 (COX-2) [25], sour cherry (0.3 g/100 g DW) had a dramatically different procyanidin profile which was dominated by short polymers, with an average chain length of four monomer units [26].

Detailed information about the health-promoting components of lesser known fruit species could lead to a better understanding of their health-beneficial effects and an increased consumption of these fruits, including their utilization in functional foods and as ingredients in nutraceuticals, medicine and pharmaceuticals [5].

There is an increased recognition and interest in utilization of lesser known fruit species not originated from the Czech Republic or neglected older fruit species, however, detailed information about the mentioned fruit species is scarce [13] because of the wide chemical diversity of antioxidant compounds present in samples of lesser known fruits [27,28,29]. Despite the presence in the literature of several papers investigating lesser known fruit species, the simultaneous determination of all above mentioned parameters is still lacking [9,30]. Moreover, all samples of non-traditional fruit species are originated from the same locality of cultivation. This fact can eliminate possible differences because of different condition of cultivation and really make possible to compare their nutritional value and find differences between them. In a similar study the phytochemical compounds of some berry species cultivated in Russia [*Rubus idaeus*, *Ribes* L. species (*R. nigrum* and *R. nigrum* × *R. dikuscha*) and the less common *Lonicera caerulea*] under the same environmental conditions were provided. *Lonicera caerulea* contained the highest level of K, *Ribes *L. presented the highest content of Ca, while *R. idaeus* displayed the highest content of Mg and different micro-elements (Fe, Mn, Zn and Mo). *Lonicera caerulea* and *Ribes* L. displayed the highest content of sugars. The most striking result was the polyphenolic contents of *L. caerulea*, which was significantly higher than that of *Ribes *L. and *R. idaeu*s, two species already known to contain large amounts of these antioxidant compounds [31]. Understanding the biochemical composition of lesser-known fruit species can stimulate an interest in maximizing their nutritional effects in human diet. The aim of this study was to determine the content of dry mater, ascorbic acid, organic acids and minerals (phosphorus, potassium, calcium, magnesium and sodium). Furthermore, we determined the phenolic profile of 11 selected genotypes of non-traditional fruit species.

## 2. Results and Discussion

Results of the chemical analyses are given in Table 1.

### 2.1. Determination of Dry Matter and Organic Acids

Results of our study given in Table 1 indicate the highest values of dry matter (DM) in *Amelanchier* ‘Smoky’ (24.0%), followed by ‘Josta’ (23.2%), and a very high content of DM was found in the new Czech cultivar of *Amelanchier* ‘NS-2’, ‘Brněnsky’ and ‘NS-1’, 23.0%, 22.4% and 22.2%, respectively [22]. According to Rivera, *et al.* dry matter content of ‘Josta’ ranged between 12–24%, which corresponded with our data [32]. 

**Table 1:** Selected nutritional parameters of cultivars/clones of non-traditional fruit species (n = 5).

Cultivars/Clones of Non-Traditional Fruit Species	Dry Matter(%)	Organic Acids(%)	ascorbic Acid (mg/100 g)	Nitrogen (mg/100 g)	Phosphorus (mg/100 g)	Potassium (mg/100 g)	Calcium (mg/100 g)	Magnesium (mg/100 g)	Natrium (mg/100 g)
*Lonicera* ‘Amur’	17.51 ± 0.87	1.46 ± 0.08	128.77 ± 2.78	402.73 ± 15.24	58.48 ± 1.40	347.71 ± 20.07	52.70 ± 0.80	14.00 ± 1.36	1.57 ± 0.05
*Lonicera* ‘Altaj’	17.20 ± 0.91	2.15 ± 0.10	133.41 ± 2.46	407.64 ± 19.31	53.66 ± 1.74	345.72 ± 21.45	41.62 ± 1.22	12.72 ± 1.06	1.89 ± 0.11
*Lonicera* ‘Sinoglaska’	13.84 ± 1.19	1.90 ± 0.16	135.11 ± 3.85	249.12 ± 17.85	39.99 ± 0.87	236.66 ± 22.23	40.82 ± 1.05	8.44 ± 0.79	2.90 ± 0.14
*Lonicera* ‘Fialka’	17.12 ± 1.10	2.57 ± 0.18	100.46 ± 2.41	383.49 ± 16.14	54.78 ± 0.97	333.84 ± 30.04	42.28 ± 1.09	13.18 ± 0.80	3.25 ± 0.24
*Lonicera *‘Goluboje vreteno’	17.32 ± 1.06	2.12 ± 0.11	111.59 ± 3.95	507.48 ± 18.24	62.00 ± 1.01	370.65 ± 24.31	42.26 ± 0.99	14.20 ± 0.95	3.63 ± 0.20
*Lonicera* ‘Amfora’	18.11 ± 0.88	2.94 ± 0.12	142.35 ± 3.74	552.36 ± 19.59	66.64 ± 0.89	421.96 ± 27.88	50.52 ± 1.79	15.39 ± 0.98	4.34 ± 0.21
L-KL-2	15.06 ± 1.11	1.58 ± 0.21	172.66 ± 13.12	185.24 ± 14.11	35.84 ± 1.10	224.39 ± 15.65	40.66 ± 0.85	10.09 ± 0.87	1.65 ± 0.11
L-KL-6	15.77 ± 0.97	1.64 ± 0.13	160.71 ± 10.12	157.70 ± 10.56	39.58 ± 1.15	252.32 ± 17.28	41.79 ± 1.00	11.82 ± 1.17	2.05 ± 0.15
L-KL-20	15.69 ± 1.02	1.88 ± 0.17	186.61 ± 8.7	243.19 ± 12.87	45.19 ± 1.17	252.61 ± 20.16	48.01 ± 1.09	12.55 ± 1.13	1.88 ± 0.08
L-KL-21	16.43 ± 1.12	1.90 ± 0.12	67.66 ± 2.54	243.16 ± 17.52	44.69 ± 1.21	254.67 ± 24.70	43.37 ± 0.90	12.65 ± 1.00	5.58 ± 0.21
L-KL-35	16.24 ± 0.84	1.87 ± 0.18	170.55 ± 9.4	215.99 ± 19.47	41.09 ± 1.50	250.09 ± 19.17	47.74 ± 1.01	11.85 ± 0.82	3.57 ± 0.22
*Morus nigra*-‘Jugoslavska’	14.60 ± 0.82	0.99 ± 0.09	40.46 ± 3.12	151.84 ± 16.45	20.00 ± 1.02	34.16 ± 3.18	14.16 ± 0.79	6.13 ± 0.75	1.46 ± 0.17
‘Josta’	23.18 ± 1.05	3.41 ± 0.20	96.80 ± 2.17	252.66 ± 18.96	56.33 ± 2.07	324.52 ± 16.10	89.01 ± 0.85	16.92 ± 0.90	2.08 ± 0.19
*Prunus tomentosa*	15.21 ± 1.01	2.56 ± 0.18	123.33 ± 3.65	147.54 ± 10.20	23.12 ± 1.19	159.71 ± 17.03	24.79 ± 1.05	6.54 ± 0.77	1.52 ± 0.09
*Amelanchier*-‘Tišnovský’	22.41 ± 1.18	1.34 ± 0.12	91.47 ± 2.48	374.25 ± 15.17	49.08 ± 1.84	356.32 ± 11.82	98.15 ± 3.44	29.80 ± 1.56	2.24 ± 0.15
*Amelanchier*-‘Brněnský’	21.38 ± 0.85	1.79 ± 0.16	110.41 ± 3.05	177.45 ± 18.62	41.26 ± 1.44	205.25 ± 9.12	67.98 ± 2.76	29.07 ± 1.47	1.71 ± 0.10
*Amelanchier*-‘Thiessen’	21.10 ± 1.20	1.40 ± 0.10	102.16 ± 4.65	244.76 ± 23.59	41.99 ± 2.03	270.08 ± 16.05	71.31 ± 2.49	22.78 ± 1.25	1.68 ± 0.18
Amelanchier-‘Smoky’	23.97 ± 1.06	1.56 ± 0.12	114.22 ± 3.88	237.30 ± 17.65	42.43 ± 1.47	294.83 ± 14.00	88.68 ± 3.51	29.00 ± 1.37	1.91 ± 0.20

Table 1 shows that the lowest values of organic acids were typical of *Morus nigra *‘Jugoslavska’ (1.0%) followed by *Amelanchier* ‘Tišnovský’ (1.3%) and *Amelanchier* ‘Thiessen’ (1.4%) [33]. The lower value of organic acid content in our study could be a result of differences in the studied cultivars and the effect of different environmental conditions, as it was reported by [22]. Similarly, in *Morus nigra* ‘Jugoslavska’ we determined a lower content of organic acids in comparison with studies of Ercisli–Orhan and Khalid *et al*. determined 1.6% acids [34,35]. What is more interesting, among all evaluated *Lonicera* samples Klčov’s clones of *Lonicera kamtschatica* exhibited the lowest content of organic acids (LKL-2 1.6% and LKL-6 1.6%). Studies by Pokorna-Jurikova *et al.* reported higher values of organic acids (2.5–4.1%) that can be accounted for by different conditions of cultivation and climatic conditions, which is supported also by previous studies in *Lonicera kamtschatica* (Sevast.) Pojark and *Lonicera edulis* ex. Freyn [10]. 

### 2.2. Determination of Ascorbic Acid

Our results indicate that the *Lonicera* clones and cultivars differ greatly in ascorbic acid values (Table 1) (67.7–186.6 mg/100 g) together with Saskatoon fruit (91.5–114.2 mg/100 g) and these values can be considered higher than in another fruit species valued for their high ascorbic acid content, such as oranges (31 mg/100 g), strawberries (46 mg/100 g) [36], kiwi fruit (29–80 mg/100 g) [37]. In our study an increased amount of ascorbic acid was observed in Klčov’s clones of *Lonicera kamtschatica*, especially LKL-20 and LKL-2 (186.6 mg/100 g; 172.7 mg/100 g), except for LKL-21 which displayed the lowest value of ascorbic acid (67.7 mg/100g). In agreement with our study, Paulovicsova *et al.* pointed to higher levels of ascorbic acid in Klčov’s clones comparable to two species—*Lonicera kamtschatica* (212.3 mg/100 g) and *Lonicera edulis* (157.5 mg/100 g). On the other hand [38] recorded a lower value of ascorbic acid in LKL-2 (30.9 mg/100 g). The statistically significant influence of different years (differences of climatic conditions) can account for differences as well [39]. The results of our study confirmed that Saskatoon berry can be considered as the second most important source of ascorbic acid (91.5–114.2 mg/100 g). The results of our studies also determined a high content of ascorbic acid in Tomentosa cherry (*Prunus tomentosa*) 123.3 mg/100 g (Table 1).

Ercisli *et al.* described moderate ascorbic acid values in genotypes of *Morus nigra* ranging from 15.1 mg/100 g to 18.7 mg/100 g [34] in comparison with our results 40.5 mg/100 g, on the other hand the studies of [38] considered black mulberry as the most important source of ascorbic acid among studied lesser known fruit species (in genotype M-103 70.5 mg/100 g). All described differences in ascorbic acid content confirmed that except for climatic conditions, the genotype also plays a very important role in ascorbic acid content as it has been reported by [40].

### 2.3. Determination of Minerals

Based on the results given in Table 1, the highest level of nitrogen was accumulated in the Russian cultivars of *Lonicera* sp., namely ‘Amfora’ 552.0 mg/100 g and ‘Goluboje vreteno’ 507.5 mg/100 g, followed by the Slovak cultivars ‘Altaj’ 407.6 mg/100 g and ‘Amur’ 402.7 mg/100 g. ‘Amfora’ also reached the leading position in phosphorus and potassium content within all evaluated less common fruit species (66.4 mg/100 g; 421.9 mg/100 g). Saskatoon berry was found to be higher in levels of calcium, magnesium, manganese compared with blueberries or strawberries. An especially high level of calcium is reported in studies of [41] (845.9 mg/kg FM in the cultivar ‘Tišnovsky’), in our study we found the highest content of calcium in this cultivar among all examined fruit species, together with the cultivar ‘Smoky’ (Ca-98.2 mg/100 g; 88.7 mg/100 g) and ‘Josta’ (89.1 mg/100 g). Similarly, we recorded the highest content of magnesium in Saskatoon berry cultivars ‘Tišnovsky’ and ‘Brněnsky’ (29.8 mg/100 g; 29.1 mg/100 g). Saskatoon berry also represented an important source of magnesium for the human diet. In Czech cultivars reached up values from 10.1 mg/kg FM (the cultivar ‘Ostravsky’) to 315.9 mgthe/kg FM (the cultivar ‘Martin’) [41], which was also confirmed in our study, where in Saskatoon berry we determined the highest values of magnesium among all evaluated less common fruit species, ranging from 22.8 mg/100 g (‘Thiessen’) up to 29.8 mg/100 g (‘Tišnovsky’). In the cultivar ‘Smoky’ we analysed lower values of calcium and magnesium content in comparison with the studies of Mazza *et al.* (111.8 mg/100 g) and a similar content of magnesium to our study (43.2 mg/100 g) [42]. 

From Table 1 it is evident that among all examined species LKL-21 exhibits the highest content of sodium (5.6 mg/100 g) followed by ‘Amfora’ (4.3 mg/100 g). 

Klčov’s clones of *Lonicera kamtschatica* formed a special group distinguished by lower values of nitrogen, phosphorus, potassium and calcium (with the exception of LKL-20) compared to other cultivars of *Lonicera*.

The results of study [10] confirmed that examined cultivars/clones of *Lonicera* displayed higher values of potassium (224.4–347.7 mg/100 g). As far as the mineral elements were concerned, the relatively high content of potassium in the cultivars ‘Thiessen’ and ‘Tišnovsky’ (4154.3 mg/kg FM and 4311.7 mg/kg FM, respectively) confirmed also the studies of [41] which are in accordance with our results. The content of magnesium (8.4–15.4 mg/100 g), calcium (40.7–50.2 mg/100 g) and sodium (1.6–4.3 mg/100 g) in berries of edible honeysuckles is lower in comparison with studies carried out under the conditions of Slovakia (71.1 mg/100 g, 107.8 mg/100 g; 8.2 mg/100 g) [10]. Lower values of mineralss can be a reflection by different conditions of cultivation and the influence of climatic conditions as it was reported by [43].

### 2.4. Determination of Polyphenolic Compounds

Finally, we determined the polyphenolic profile of 11 genotypes (selected clones and cultivars) of non-traditional fruit species (Table 2), especially the content of gallic acid, catalposide, rutin, resveratrol, quercitrin and chlorogenic acid with quercetin was examined.

From all evaluated samples the highest content of gallic acid and catalposide was determined in Saskatoon berry ‘Smoky’ (116.5 mg/100 g, 85.6 mg/100 g), followed by black mulberry ‘Jugoslavska’ (95.6 mg/100 g, 75.9 mg/100 g), while samples of edible honeysuckle reached values of gallic acid of up to 15.01 (LKL-2)–39.5 mg/100 g (LKL-35) (Table 2). *Amelanchier alnifolia* reached a comparable gallic acid content as *Amelanchier* ‘Thiessen’ [20]. Rutin predominated in ‘Thiessen’ 93.3 mg/100 g (Table 2) having four times higher values in comparison with *Amelanchier alnifolia* [11] also LKL-35 87.9 mg/100 g can be considered as reach source of rutin. We measured a similar concentration of resveratrol in all samples of lesser-known fruit species, except for ‘Smoky’ (6.5 mg/100 g).

**Table 2:** Polyphenolic profile of selected cultivars/clones of non-traditional fruit species in mg/100 g (n = 5).

Genotyp	Gallic acid	Catalposide	Rutin	Resveratrol	Quercitrin	Chlorogenic acid	Quercetin
Amur	22.68 ± 1.03	45.38 ± 1.23	43.31 ± 0.7	1.88 ± 0.05	12.60 ± 1.10	153.37 ± 10.01	15.76 ± 0.9
Altaj	19.85 ± 0.97	28.59 ± 0.5	15.17 ± 1.37	1.59 ± 0.11	N.D.	86.62 ± 0.89	12.15 ± 1.03
Sinoglaska	26.33 ± 0.92	25.42 ± 1.02	18.64 ± 1.01	1,67 ± 0.13	N.D.	140.54 ± 10.56	13.37 ± 0.75
Amfora	23.28 ± 0.75	30.19 ± 1.47	35.97 ± 2.1	N.D.	10.057 ± 0.86	100.81 ± 8.02	14.11 ± 0.92
LKL-2	15.01 ± 1.3	22.87 ± 1.24	27.54 ± 0.75	1.62 ± 0.12	12.45 ± 1.11	N.D.	N.D.
LKL-20	28.23 ± 1.18	N.D.	54.73 ± 2.04	2,09 ± 0.05	6.27 ± 0.74	172.99 ± 11.5	15.42 ± 0.93
LKL-21	27.69 ± 1.20	N.D.	43.48 ± 3.1	N.D.	5.68 ± 0.2	130.18 ± 5.6	14.17 ± 1.0
LKL-35	39.45 ± 0.55	N.D.	87.92 ± 3.4	N.D.	N.D.	267.14 ± 12.7	8.44 ± 0.64
*Morus* Jugoslavska	95.6 ± 2.3	75.94 ± 0.97	26.09 ± 1.17	1.95 ± 0.08	14.29 ± 1.03	245.43 ± 17.3	13.23 ± 1.01
*Amelanchier* Thiessen	24.31 ± 1.06	N.D.	64.62 ± 2.76	N.D.	12.36 ± 1.13	298.13 ± 15.6	23.52 ± 1.52
Amelanchier Smoky	116.49 ± 2.42	85.56 ± 3.2	N.D.	6.53 ± 0.22	N.D.	N.D.	30.68 ± 1.61

Quercitrin levels above 10 mg/100 g were determined in black mulberry ‘Jugoslavska’ (14.3 mg/100 g), ‘Amur’ (12.6 mg/100 g) and ‘Thiessen’ (12.4 mg/100 g) (Table 2) but all samples of non-traditional fruit species had lower values in comparison with the results of [11].

Chlorogenic acid was the major examined polyphenolic compound in all samples with the highest levels being found in Saskatoon berry ‘Thiessen’ (298.1 mg/100 g) and LKL0-35 (267.1 mg/100 g) and finally in black mulberry ‘Jugoslavska’ (245.4 mg/100 g) (Table 2). Both analysed cultivars of Saskatoon berry can be characterised by exceptionally high value of quercetin ‘Thiessen’ 23.6 mg/100 g, ‘Smoky’ 30.7 mg/100 g (Table 2). The authors of [20] determined in berries of cultivar *Lonicera * ‘Altaj’ 60 mg/100 g gallic acid, 24 mg/100 g rutin and 22 mg/100 g quercitrin, but our experiments indicated lower values of polyphenolic compounds. According to Jurikova *et al. *the level of rutin obtained at several genotypes of *Lonicera *spp. does not surpass a level of 30 mg/100 g [10] but in our experiments we saw higher concentrations in ‘Amur’, ‘Amfora’, LKL-20, LKL-21 and LKL-35 [11]. In ‘Sinoglaska’ 59.8 mg/100 g gallic acid, 24.3 mg/100 g rutin and 22.2 mg/100 g qurcetin was found and in LKL-21 69.2 mg/100 g gallic acid, that represented lower values in comparison with our values, but the level of rutin in the mentioned genotype is higher. The concentration of gallic acid in ‘Thiessen’ is similar to Saskatoon berry ‘Martin’, reaching up to 27.7 mg/100 g, but the concentration of rutin in ‘Thiessen’ was two times higher. This fact can be caused by differences in stage of ripeness, and conditions of storage as it has been mentioned by intraspecific diversity of samples, [44,45], or the different procedures used in the extraction of samples [44,46]. 

### 2.5. Statistical Evaluation of Results

On the basis of statistical evaluation of data according to dry matter, organic acids and ascorbic acid content the non-traditional fruit species (cultivars/clones) can be separated into three individual clusters.

The first cluster was represented by *Lonicera* ‘Fialka’, LKL-21, *Morus nigra* ‘Jugoslavska’, ‘Josta’, *Amelanchier *‘Tišnovský’ and *Amelanchier* ‘Thiessen’. The second cluster was formed by *Lonicera *‘Amur’, *Lonicera *‘Altaj’, *Lonicera* ‘Sinoglaska’, *Lonicera* ‘Goluboje vreteno’, *Lonicera *‘Amfora’, *Prunus tomentosa*, *Amelanchier *‘Brněnský’, Amelanchier ‘Smoky’. The third cluster included Klčov’s clones: LKL-2, LKL-6, LKL-20 and LKL-35. Similarly, according to mineral matter content cluster analysis separated three individual clusters:

1. Cluster: *Lonicera* ‘Sinoglaska’, LKL-2, LKL-6, LKL-20, LKL-35, *Morus nigra* ‘Jugoslavska’, *Prunus tomentosa*;
2. Cluster: ‘Josta’, *Amelanchier*-‘Tišnovský’, Amelanchier-‘Brněnský’, Amelanchier-‘Thiessen’, Amelanchier ‘Smoky’;
3. Cluster: *Lonicera* ‘Amur’, *Lonicera* ‘Altaj’, *Lonicera *‘Fialka’, *Lonicera *‘Goluboje vreteno’, *Lonicera* ‘Amfora’and LKL-21.

## 3. Experimental

### 3.1. Description of Growing Locality

Fruits were harvested in an experimental gene-fund orchard of Mendel University in Brno over the may-september period in 2008. This orchard is situated in the area of the village called Zabcice, approximately 20 km south of Brno, in the Czech Republic. The altitude is 184 m. The average annual temperature and a fifty-year average sum of precipitation are 9 °C (during the growing season 15.6 °C) and 553 mm (during the growing season 356 mm), respectively. The soils are classified as gleyed alluvial soils developed on the Holocene calciferous sediments with a marked accumulation of organic compounds. As far as the texture is concerned, the topsoil is loamy and the subsoil clayey-loamy.

### 3.2. Biological Material

The following less common fruit samples we used: six cultivars of *Lonicera *spp. originated in Slovakia (Research Institut of Fruit and Decorative Trees in Bojnice) from free pollination of Gerda (‘Amur’) and the crossing of *Lonicera kamtschatica* X *Lonicera turczaninowii* (‘Altaj’), four cultivars come from the territory of Russia-‘Goluboje vreteno’ as seedlings obtained from free pollination of *Lonicera kamtschatica* having origin in the “Sady Sibiri” orchards, ‘Amfora’ and ‘Fialka’, ‘Goluboje vreteno’ and ‘Sinoglaska’ are seedlings of the cultivar ‘Roksana’ selected in Pavlovsk Research Institut. Five clones of *Lonicera kamtschatica* (Sevast.) Pojark., namely LKL-2, LKL-6, LKL-20, LKL-21 and LKL-35 were selected from the same hybrid seeds from Lisavenko State Research Institute of Siberian Horticulture by breeders of Herbaton Klčov s.r.o. Saskatoon berry (*Amelanchier alnifolia* Nutt.) was represented in four cultivars: ‘Smoky’, ‘Thiessen’, ‘Brněnsky’ and ‘Tišnovsky’. ‘Tišnovsky’ and ‘Brněnsky’ are ecotypes from the selection in the Czech Republic, ‘Thiessen’ and ‘Smoky’ are from Canada [40]. Jostaberry-‘Josta’ is a cross between *Ribes nigrum *and *Grossularia uva*-*crispa.* Black mulberry ‘Jugoslavska’ is an ecotype of *Morus nigra*. 

The polyphenolic profile was examined in 11 genotypes-*Lonicera*-‘Amur’, ‘Altaj’, ‘Sinoglaska’, LKL-2, LKL-20, LKL-21, LKL-35, *Morus nigra* ‘Jugoslavska’, *Amelanchier* ‘Thiessen’ and ‘Smoky’.

### 3.2. Collection of Samples

Fruit were harvested from five plants of each cultivar/clone under study within the period of consumption ripeness. Harvested fruits were puréed in a mixer and an averaged sample was obtained by dividing into quarters. Each parameter was measured in five replicates. Twenty randomly chosen fruits from each tree/shrub were used for analyses (*i.e.*, altogether 100 per each cultivar/clone). For determination of ascorbic acid content samples of fruit were processed immediately after the harvest. For analyses of another parameters samples were stored at −18 °C for six month.

### 3.3. Chemical Analyses

#### 3.3.1. Determination of Dry Matter and Organic Acids

The content of the dry matter was determined by drying at 105 °C ± 2 °C. The amount of acids (titrable acidity) was determined in aqueous extract with potentiometer by titration of sodium hydroxide (c = 0.1 mol·dm^−3^) at the pH level = 8.1. The content of organic acids was expressed in g/100 g of fresh weight.

#### 3.3.2. Mineral Content Assay

To determine the content of mineral elements assay the sample was dried to a constant weight in a drier at 105 °C ± 2 °C; thereafter, 1 g of homogenised dry matter (with the 1-mm size particles) was further mineralised in a mixture of concentrated sulphuric acid with 30% hydrogen peroxide. After the mineralization, the obtained samples were quantitatively transferred into a 250-mL volumetric flask and filled to the volume with re-distilled water. The resulting mineralisate was measured in an atomic absorption spectrometer (PHILIPS PU 9200X). The amount of crude protein was estimated on the base of total nitrogen in a KJELTEC TM 2300 Kjeldahl apparatus and the result was multiplied by the coefficient 6.3. The content of mineral elements was expressed in mg/100 g of fresh weight (in case of crude protein as g/100 g of fresh weight).

#### 3.3.3. Determination of Ascorbic Acid

Determination of ascorbic acid content was carried out by a modification of the methods by Wagner *et al*. [47] and Miki [48]. Sample (5 g) was weighed in an Erleymayer flask and 25 mL of extractant methanol:H_2_O:H_3_PO_4_ in the rate 99:0.5:0.5 were added. The flask with samples was placed into a water bath at a temperature of 25 °C whereby samples were extracted for 15 min. To keep the samples out of daylight the flask was covered by aluminium foil during the preparation. After the extraction the content of the bank was filtered through paper Filtrapak No. 390. The filtrate prepared in this way before injection was diluted in a portion of extractant and filtered again through a nylon membrane filter (0.45 μm nylon filter disk). The instrument used for ascorbic acid analysis consisted of a solvent delivery pump (ESA Inc., Model 582), guard cell (ESA Inc., Model 5010A, working electrode potential K1 = 600 mV, K2 = 650 mV), chromatographic column (Model Supelcosil LC8 (150.0 × 4.6 mm), 5 µm particle size) and an electrochemical detector (Coulochem III). Chromatographic conditions were constant: 30 °C, as a mobile phase methanol was used: H_2_O:H_3_PO_4_ = 99:0.5:0.5, (filtered through a nylon filter 0.2 µm), the type of elution was isocratic, the flow rate of the mobile phase was 1.1 mL/min, retention time 1.9–2.0 min. The content of ascorbic acid was calculated on fresh weight mg/100 g.

#### 3.3.4. Laboratory Equipment and Instruments for Determination of HPLC Profile (HPLC-ED)

For examination of HPLC profile-gallic acid, catalposide, rutin, resveratrol, quercitrin, chlorogenic acid and quercetin the HPLC-ED technique was used. HPLC-system I: The HPLC-ED instrument consisted of one solvent delivery pump (Model 582 ESA Inc., Chelmsford, MA, USA), a guard cell (Model 5020 ESA Inc.), chromatographic column Restec Allure reverse phase column (150.0 × 4.6 mm, 5 μm particle size; Belefonte, PA, USA), and the electrochemical detector. The amperometric detector includes one low volume flow-through analytical cells (Model 5040, ESA Inc.) consisting of a glassy carbon working electrode, hydrogen-palladium electrode as reference electrode and auxiliary carbon electrode, and Coulochem III as a control module. The samples (5 μL) were injected using autosampler (Model 540, ESA Inc.). The data were treated by CSW 32 software (Version 1.2.4; Data Apex, Prague, Czech Republic). Guard cell potential was set as 0 V. A glassy carbon electrode was polished mechanically by 0.1 μm of alumina (ESA Inc.) and sonicated at room temperature for 5 min using a Sonorex Digital 10 P Sonicator (Bandelin, Berlin, Germany) at 40 W. HPLC-system II: chromatographic pump Model 582 (ESA Inc.), autosampler Model 542 ESA (ESA Inc.), vacuum desaser (Shimadzu Corp., Duisburg, Germany), chromatographic column with reverse phase Zorbax C 18-AAA (150 × 4.6 mm, 4.6 mm particle size 3.5 μm, Agilent Technologies, ESA Inc., Santa Clara, CA, USA), 12-channels electrochemical detector CoulArray (Model 5600A, ESA Inc.) with flow-through analytical cell (Model 6210, ESA Inc.). Chromatographic conditions were optimized according to following parameters: the volume of injection was 30 µL, mobile phase A consisted of formic acid 0.2% (v/v), MP B was acetonitrile. The gradient composition was linearly increased from 12 up to 22% for mobile phase B (v/v) for 2 minutes from start, to 50% for 25 min, to 55% (30 min). Separation process was inducted by eluation with the negative gradient at 15% B for 45 min. Flow rate was 0.8 µL/min. The electrochemical detector scanned responses at applied working electrode potentials of −80, 0, 80, 160, 240, 320, 400, 480, 560, 640, 720 and 800 mV. The resulting detection was expressed in microcoulombs.

#### 3.3.5. Statistical Evaluation

Statistical evaluation and separation of non-traditional fruit species-their clones and cultivars were provided by cluster analysis in the *Statistica *programme on the basis of K-mean values after standardization of dates.

## 4. Conclusions

In this study it is shown that there were marked differences in the selected nutritional values of non-traditional fruit species grown in Žabčice-genofond belonging to MENDELU Brno. *Lonicera* cultivars/clones had high levels of ascorbic acid and mineral elements, especially the cultivar ‘Amfora’ which reached the leading position in nitrogen, phosphorus and potassium content among all studied lesser known fruit species. *Amelanchier* cultivars had the second position in ascorbic acid content and also represented a valuable source of calcium; particularly the cultivars ‘Tišnovský’ and ‘Smoky’ accumulated the highest level of this mineral among all examined species. Both cultivars of *Amelanchier*—‘Thiessen’ and ‘Smoky’—together with *Morus nigra* ‘Jugoslavska’ represented the most valuable source with respect of examined polyphenolic compounds. We can conclude that the consumption of less common fruit species can bring health benefits and have a high potential value for fruit growers as well as nutraceutical manufacturers. Regarding the fact that all cultivars were grown under identical conditions and in the same locality, it is possible to conclude that one can clearly see the cultivar variability, which is quite typical of non-traditional fruit.
